# Pneumococcal Encounter With the Blood–Brain Barrier Endothelium

**DOI:** 10.3389/fcimb.2020.590682

**Published:** 2020-11-03

**Authors:** Anjali Anil, Anirban Banerjee

**Affiliations:** Department of Biosciences and Bioengineering, Indian Institute of Technology Bombay, Mumbai, India

**Keywords:** meningitis, *Streptococcus pneumoniae*, blood-brain barrier, brain microvascular endothelial cells, transcytosis, autophagy, ubiquitin-proteasome machinery

## Abstract

Meningitis, the inflammation of the protective membrane surrounding the brain and spinal cord (known as meninges), is a condition associated with high mortality rates and permanent neurological sequelae in a significant proportion of survivors. The opportunistic pathogen *Streptococcus pneumoniae* (SPN/pneumococcus) is the leading cause of bacterial meningitis in adults and older children. Following infection of the lower respiratory tract and subsequent bloodstream invasion, SPN breaches the blood–brain barrier endothelium for invasion of the central nervous system. Transcytosis, a mode of passage through the endothelial cells has been identified as the predominant route of pneumococcal blood–brain barrier trafficking. Herein, we review the interactions enabling SPN invasion into the brain endothelial cells, events involved in the tug-of-war between pneumococcal virulence factors and host intracellular defense machineries and pneumococcal strategies for evasion of host defenses and successful transendothelial trafficking.

## Introduction

The central nervous system (CNS) consists primarily of the brain, the control center of the body, and the spinal cord, enveloped in meninges and protected by the skull and vertebrae, respectively. The meninges is a membranous covering of connective tissue whose primary function is to shield the brain and spinal cord from trauma. It is composed of 3 layers: outermost dura matter, arachnoid, and the inner pia matter. The space between the arachnoid and pia matter is called the subarachnoid space and houses the cerebrospinal fluid and major vasculature. Acute inflammation of the meninges, a condition known as meningitis, is triggered by certain infections, autoimmune disorders, cancer, and drugs and is associated with a high mortality rate and long-term neurological sequelae in survivors (Collaborators, 2018). *Streptococcus pneumoniae, Neisseria meningitidis*, and *Hemophilus influenzae* are the major etiological agents of bacterial meningitis in adults and older children, and group B streptococcus is responsible for most cases in neonates (Oordt-Speets et al., 2018). *Streptococcus pneumoniae* (SPN/pneumococcus), a commensal resident of the human nasopharynx and an opportunistic pathogen, causes meningitis following bloodstream invasion from the lower respiratory tract and high-grade bacteremia (Mook-Kanamori et al., 2011). Although a spread of infection directly from the middle ear (following otitis media) to the brain has been reported (Marra and Brigham, 2001), SPN predominantly adopts the hematogenous route and breaches the blood–brain barrier (BBB) for invasion of the CNS.

The BBB is constituted by brain microvascular endothelial cells (BMECs), which form the wall of the blood capillaries, maintained by support from the basement membrane, astrocytes, and pericytes and helps to maintain homeostasis of the CNS. The BBB endothelium is characterized by the presence of tight junctions and low pinocytosis/transcytosis ability. The tight junctions composed of claudins (specifically, claudin-3, −5, and −12), occludins and junction adhesion molecules are present toward the apical (luminal) side of the barrier and, along with the adherens junctions, contributes to the high transendothelial electrical resistance and dictates polarity to the BBB endothelium (Sandoval and Witt, 2008; Luissint et al., 2012). These features, along with the asymmetric distribution of efflux and nutrient transporters across the polarized endothelial membrane, enable it to strictly control the transport of blood-borne molecules into the brain and guard the latter from harmful materials present in the circulatory system (Daneman and Prat, 2015). Meningeal pathogens, however, have been shown to breach the BBB by (a) paracytosis: traversal via the intercellular space, (b) transcytosis: intracellular trafficking through the endothelial cells, or (c) Trojan horse mechanism: utilizing infected phagocytes as vehicles (Doran et al., 2013). Apart from these strategies, recognition of pathogen-associated molecular patterns (PAMPs) by host pattern recognition receptors (PRRs) following microbial invasion and replication in the brain elicits an overwhelming inflammatory response. Ensuing leukocyte recruitment along with the combined cytotoxicity of microbial toxins and reactive oxygen/nitrogen species generated by the immune cells lead to BBB disruption, which additionally fosters pathogen infiltration into the brain.

Events involved in the interaction of SPN with the BBB leading to meningitis is studied in detail by Iovino et al. (2013) using a clinical meningitis isolate TIGR4 (serotype 4) in a mouse model of bacteremia-derived meningitis. Subarachnoid vessels were identified as the primary contact site of SPN with the brain (at 1 h post-infection), which later spread to the cerebral cortex, septum, and eventually, to choroid plexus (by 8 h post-infection). Early in the course of infection, SPN were found tightly attached to the BBB endothelium; however, these numbers reduce with time with a concomitant increase in the number of SPN present in the brain tissue, suggesting pneumococcal translocation across the BBB. Interestingly, the junctions between the endothelial cells in the subarachnoid space and choroid plexus were found to be intact during the course of infection, suggesting that SPN predominantly adopts the transcytosis route for crossing the BBB, especially in the early stages of infection. Indeed, a follow-up study confirmed the presence of SPN inside the BMECs *in vivo* (Iovino et al., 2014). In this review, we summarize the molecular events involved in pneumococcal interaction with the BMECs, facilitating its trafficking across the BBB.

### Adherence and Invasion

**Figure 1:**
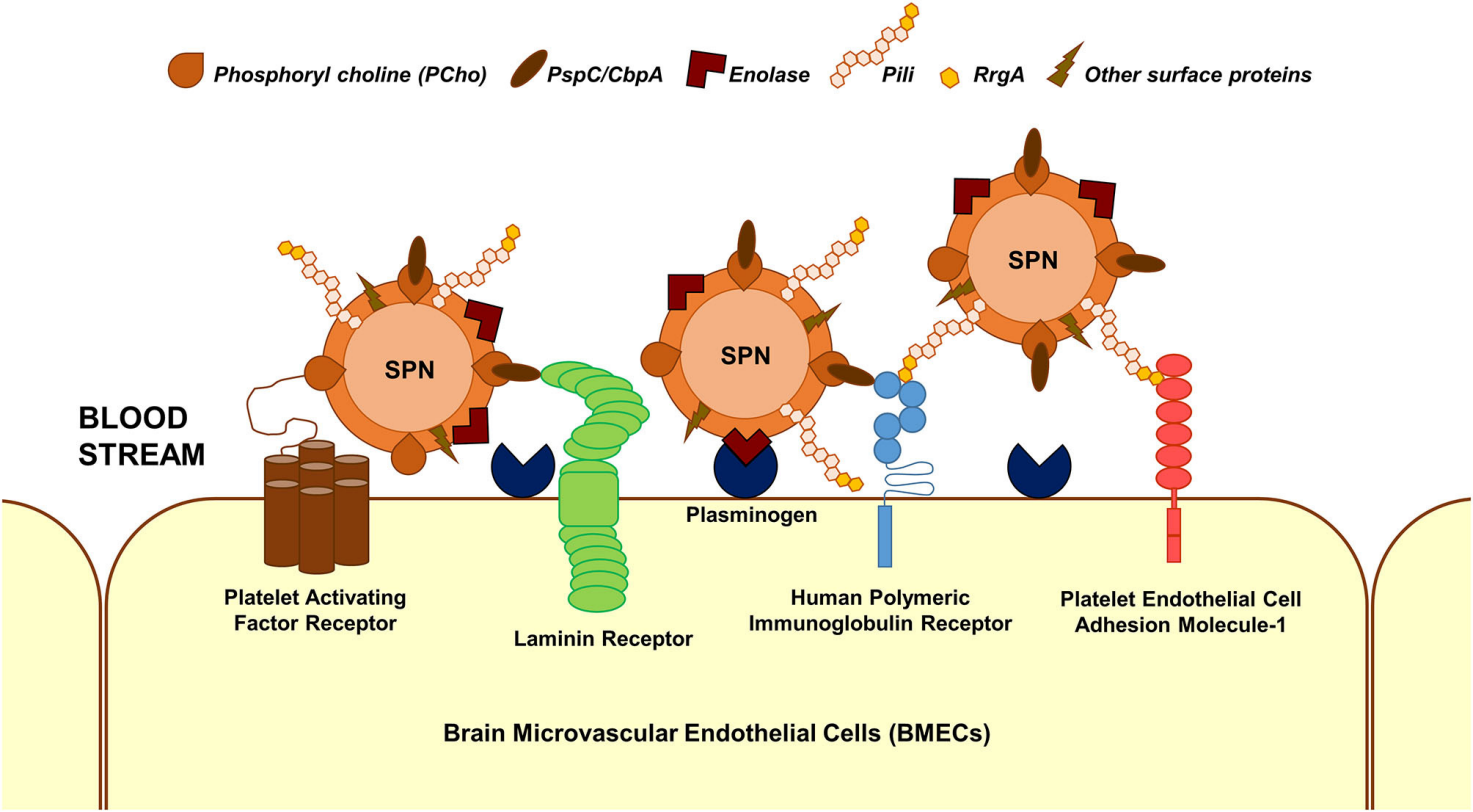
Major interactions facilitating SPN adhesion to BMECs. Major interactions between SPN ligands and host cell receptors that enable adhesion of blood-borne SPN to BMECs are depicted. These involve but are not limited to phosphoryl choline–platelet activating factor receptor, PspC (CbpA)–laminin receptor, PspC–human polymeric immunoglobulin receptor, RrgA (component of pneumococcal pili)–human polymeric immunoglobulin receptor, RrgA–platelet endothelial cell adhesion molecule 1, and enolase–plasminogen interactions.

The first step in the interaction of blood-borne SPN with brain endothelium is adherence/attachment to BMECs. Multiple interactions between pneumococcal surface proteins and the brain endothelial cell receptors are known to facilitate this (Figure 1); some of these interactions are common across different cell types. Additionally, some of the receptors can bind to multiple ligands on the SPN surface and vice versa. The laminin receptor on the BMECs serve as a common receptor for the attachment of a wide array of meningeal/neurotropic agents (Orihuela et al., 2009). SPN interacts with the laminin receptor via PspC (also called CbpA), a member of the family of choline-binding proteins, which anchor to phosphoryl choline on the SPN cell wall (Orihuela et al., 2009). PspC additionally facilitates SPN adhesion to brain endothelium via its interaction with the human polymeric immunoglobulin receptor (hpIgR) (Iovino et al., 2017). hpIgR, along with the platelet endothelial cell adhesion molecule (PECAM-1), facilitate binding of SPN via the pneumococcal pilus-1 adhesin RrgA (Iovino et al., 2017). Enolase, the pneumococcal glycolytic enzyme is a moonlighting protein that gets secreted via an unknown mechanism (Bergmann et al., 2001). The surface-displayed enolase acts as a receptor to host cell surface-bound plasminogen, promoting SPN adherence to BMECs (Bergmann et al., 2013). A recent study that utilized a proteomics-bioinformatics approach to identify SPN cell wall ligands that mediate adherence to BMECs revealed 5 putative candidates: adhesion lipoprotein, pneumococcal histidine triad protein A (PhtA), endo-β-N-acetylglucosaminidase, and two hypothetical proteins: Spr0777 and Spr1730 (Jimenez-Munguia et al., 2018). Their respective interacting partners on the endothelium, however, remains to be investigated.

Following adherence, SPN internalize into (invade) endothelial cells via the endocytic pathway. Endocytosis is a eukaryotic cellular process designed for uptake of nutrients from the extracellular milieu but is exploited by microbes to gain entry into host cells (Cossart and Helenius, 2014). SPN have been shown to enter the BMECs via multiple endocytic routes, specifically clathrin- and caveolae-mediated pathways (Gradstedt et al., 2013). The former is characterized by the formation of clathrin coats on the vesicles budding off from the plasma membrane, and the latter is distinguished by the presence of caveolin and formation of flask-shaped invagination at cholesterol-rich domains of the plasma membrane (lipid rafts). Both of these are classified under dynamin-dependent pathways, which utilize the GTPase dynamin for the scission of vesicles from the plasma membrane. Recent findings from our lab additionally demonstrate a role of dynamin-independent pathways in facilitating SPN entry into BMECs (Surve et al., 2020).

One of the earliest identified SPN–host cell interactions is the binding of phosphoryl choline (PCho) present on SPN cell walls to the platelet-activating factor receptor (PAFR) on epithelial and endothelial cells (Cundell et al., 1995). This molecular mimicry of platelet-activating factor by PCho for binding to PAFR is also demonstrated by other respiratory/meningeal pathogens, such as *Neisseria meningitidis* and *Hemophilus influenzae* (Swords et al., 2001; Jen et al., 2013). PCho-PAFR interaction not only fosters SPN adherence to host cells, but also internalization via the clathrin-dependent endocytosis involving the adapter molecule β-arrestin 1 (Radin et al., 2005). Inflammatory activation of BMECs upon treatment with tumor necrosis factor (TNF-α) is demonstrated to remarkably improve SPN invasion via upregulation of PAFR expression (Cundell et al., 1995). Additionally, the transparent phase variants of SPN that harbor more PCho on the surface invades BMECs significantly better than the opaque phase variants (Ring et al., 1998). Neuraminidase A (NanA) is a surface-attached exoglycosidase that removes terminal sialic acid from glycoconjugates, in turn, serving diverse purposes, such as nutrient acquisition, unmasking host receptors for attachment, disabling host components involved in bacterial clearance, etc. (King et al., 2006). Additionally, NanA has been demonstrated to induce inflammatory activation of BMECs in a sialidase-independent, laminin G–like lectin binding domain-dependent manner, enabling improved invasion of BMECs (Banerjee et al., 2010). Although ligand-receptor interaction facilitating SPN uptake into BMECs via a caveolae-dependent pathway has not been identified, the PspC-hpIgR interaction is shown to mediate SPN internalization via both clathrin- and caveolae-dependent endocytosis in epithelial cells (Asmat et al., 2014).

The polysaccharide capsule of SPN is an antiphagocytic factor that provides an advantage during bloodstream invasion by enabling it to escape complement-mediated opsonophagocytosis (Hyams et al., 2010). Presence of the capsule, however, poses a problem during SPN interaction with non-phagocytic cells and is shown to impede invasion of BMECs (Ring et al., 1998). To overcome this, SPN downregulate expression of the capsule during interaction with epithelial cells both *in vitro* and *in vivo*, enabled by the controlled activity of cell wall amidase LytA (Hammerschmidt et al., 2005; Kietzman et al., 2016). However, preliminary studies that compare the association of SPN with BBB endothelium using antipneumococcal serum and an antibody against the polysaccharide capsule reveal no difference, suggesting that the capsule is likely maintained during pneumococcal interaction with BMECs (Iovino et al., 2013).

Candidate combox site 4 (ccs4) is a competence-induced protein that has been implicated in facilitating SPN association and invasion of BMECs although the details of this interaction remain to be investigated (Hirose et al., 2018). With a plethora of SPN factors working in concert to aid SPN invasion of the CNS, a member of the family of paralogous zinc metalloproteases, ZmpC, has been demonstrated to impede BMEC invasion (Yamaguchi et al., 2017). This counterintuitive function of ZmpC is thought to be an evolutionary adaptation in SPN, aimed at attenuating virulence in order to minimize host mortality, in turn, enabling prolonged infection and replication within the host (Yamaguchi et al., 2017).

### Intracellular Fate

Transmission electron microscopy analysis of infected BMECs reveals that SPN resides within intracellular vacuoles (Ring et al., 1998). The endocytic vacuole formed following fission of the plasma membrane undergoes a maturation process (from early to late endosome), acquiring different protein (Rab GTPases and effectors) and lipid markers (phosphoinositides) and eventually fuses with lysosomes resulting in the degradation of the cargo that they carry (Huotari and Helenius, 2011). Endosomal maturation is also accompanied by progressive acidification of the vacuolar lumen, which is critical for the optimum activity of lysosomal hydrolases. A major fraction of SPN internalized into BMECs following PCho-PAFR interaction associated initially with Rab5 or EEA1 (markers of the early endosome) and later with Rab7 or LAMP-1 (markers of late endosome or lysosome), suggesting that they proceed toward lysosomal degradation (Radin et al., 2005). Indeed, treatment of BMECs with ammonium chloride (NH_4_Cl) or chloroquine, which inhibit lysosome acidification, results in improved intracellular survival of SPN (Gradstedt et al., 2013). Inhibition of lysosomes also correlates with improved ability of SPN to transcytose across the BMECs in an *in vitro* transcytosis assay (Gradstedt et al., 2013). On the other hand, overexpression of β-arrestin 1, the protein involved in PAFR-mediated uptake of SPN, is found to reduce colocalization of SPN containing vacuoles with Rab7, suggesting its role in shunting of the vacuoles away from lysosomal degradation (Radin et al., 2005). The recently identified dynamin-independent pathway of pneumococcal internalization is also found to prevent targeting of SPN containing vacuoles to lysosomes (Surve et al., 2020).

Apart from the endocytic pathway, few other cellular homeostasis pathways also function as defense machineries against intracellular microbes; examples of these include autophagy and ubiquitin-proteasome machineries, which normally function to degrade aged organelles and damaged/misfolded proteins. A recent study reveals that SPN interacts with these pathways inside BMECs in a manner dependent on the expression of its pore-forming toxin pneumolysin (Surve et al., 2018). Pneumolysin (Ply) belongs to the family of cholesterol-dependent cytolysins (CDCs) and forms pores on eukaryotic membranes by a 3-step process consisting of (a) monomer binding to membrane cholesterol, (b) oligomerization to form a pre-pore structure, and (c) pre-pore to pore transition (Tilley et al., 2005). Surve et al. (2018) demonstrate that Ply expressed by SPN within BMEC vacuoles creates pores and ruptures the vacuolar membrane. This damage is sensed by the host cytosolic “eat me” signal galectin-8 (Gal8), which then binds to exposed glycans on the luminal side of ruptured vacuoles and interacts with the adapter molecule NDP52 to trigger antibacterial autophagy (xenophagy). Induction of autophagy results in enveloping of damaged SPN containing vacuoles in double membrane bound structures (autophagosomes) decorated with LC3B for fusion with lysosomes. Treatment with 3-methyl adenine, an autophagy inhibitor, improved the intracellular survival of SPN confirming the role of autophagy in SPN killing within BMECs (Surve et al., 2018). Interestingly, a subset of SPN is observed to reside within non-acidified autophagosomes for a prolonged period of time; however, the factors governing this phenotype remain elusive (Surve et al., 2018). In this context, CbpC (a choline-binding protein) released by SPN is shown to interact with and direct Atg14 (an autophagy-related protein) toward autophagic degradation in epithelial cells and fibroblasts, thus utilizing Atg14 depletion as a strategy to subvert xenophagic killing of intracellular SPN (Shizukuishi et al., 2020). Furthermore, excessive damage to the vacuolar membrane by Ply pores allows exit of SPN from the vacuole into the cytosol. Although cytosolic escape is advantageous for certain pathogens, such as *Listeria monocytogenes*, and facilitates cell-to-cell spread (Schnupf and Portnoy, 2007), cytosolic SPN is found to be recognized and tagged by host ubiquitin (Ubq) machinery for degradation either via autophagy or an autophagy-independent, proteasome-dependent pathway (Surve et al., 2018). Recent studies exploring the molecular details of SPN ubiquitination inside epithelial cells reveals that cytosolic SPN and vacuolar membrane remnants are tagged with the K48-type Ubq chains for autophagic degradation while SPN-containing autophagosomes harbor the K63-type Ubq chains, formed by the action of Nedd4-1 E3 ligase (Ogawa et al., 2018). Additionally, SPN is also shown to associate with the proteasome inside BMECs *in vivo* (Iovino et al., 2014), and proteasome inhibition by treatment with MG132 improves SPN intracellular survival (Iovino et al., 2014; Surve et al., 2018).

Studies by Surve et al. (2018) further illustrate the simultaneous existence of 6 different subsets of SPN within BMECs, each characterized by their association with a different combination of degradative pathway markers (Gal8, Ubq, and LC3). These intracellular subsets are found to arise as a consequence of heterogeneous expression of Ply among the individual cells of an isogenic SPN population (Surve et al., 2018). SPN expressing a low amount of Ply (SPN:Ply-low) are found to be predominantly confined to the vacuole while those expressing a high amount of Ply (SPN:Ply-high) are mostly cytosolic with the former demonstrating improved intracellular survival compared to the latter (Surve et al., 2018). SPN:Ply-high, owing to extensive damage to vacuolar membrane became cytosol exposed where they were detected by host ubiquitin machinery and subjected to clearance (Surve et al., 2018). SPN:Ply-low also occupied a unique vacuole, devoid of Gal8 and Ubq but positive for LC3. These Gal8^−^Ubq^−^LC3^+^ vacuoles are speculated to originate as a result of formation of small ion-channel-sized pores on the vacuolar membrane (by action of low amount of Ply), causing osmotic imbalance and, in turn, recruiting LC3 independent of conventional autophagy markers (a form of non-canonical autophagy) (Florey et al., 2015; Surve et al., 2018). A similar, Ply-dependent formation of non-canonical, LC3-associated phagosome (LAP)-like vacuoles harboring SPN has also been observed in epithelial cells and were found to serve as a precursor to the formation of canonical SPN containing autophagosomes (Ogawa et al., 2020). The improved ability of SPN:Ply-low to survive inside BMECs are also reflected in their ability to transcytose across the BBB and invade the brain in a mouse model of meningitis (Surve et al., 2018). Consistent with these findings, clinical meningitis isolates TIGR4 (serotype 4) and Tupelo (serotype 14) were found to consist of low numbers of Ply producers in comparison to a sepsis strain D39 (serotype 2) or a colonizer strain A60 (serotype 19F) (Surve et al., 2018).

### Recycling and Transcytosis

Although a major fraction of SPN get killed by the machineries inside the host cell, a subset successfully evade the intracellular defenses and transcytose across the endothelium for invasion of the brain, resulting in uncontrolled bacterial replication and development of meningitis. Another fraction was found to recycle out of apical side of the endothelium in vacuoles decorated with Rab11 (marker of recycling endosome) (Radin et al., 2005). This population, which recycles back into the bloodstream, is thought to serve as a reservoir for infection, temporarily hidden from the host intracellular defenses (Ring et al., 1998).

The opaque and transparent phase variants demonstrate a clear distinction in their intracellular fates. The opaque variants are mostly killed within the BMECs while the transparent variants undergo transcytosis (Ring et al., 1998). A fraction of transparent variants also recycle back to the apical surface (Ring et al., 1998). PAFR-mediated invasion and association of β-arrestin 1 with SPN containing vacuoles is suggested to play a role in this by reducing its association with Rab7 and skewing SPN fate toward transcytosis away from lysosomal killing or recycling (Ring et al., 1998; Radin et al., 2005). There exists another school of thought according to which transcytosis across endothelial cells is mediated by caveolae-dependent endocytosis (Simionescu et al., 2009). Whether the PspC-hpIgR interaction leads to caveolae-dependent invasion in endothelial cells and whether this pathway supports SPN transcytosis is yet to be explored.

The low Ply expressing SPN subset demonstrates improved transcytosis ability both *in vitro* and *in vivo* (Surve et al., 2018). Pore-forming toxin mediates release of Ca^2+^ from bacteria-containing vacuoles into the host cytosol has been speculated to facilitate exocytosis-like exit of *Serratia marcescens* from the host cell (Di Venanzio et al., 2017); a similar mechanism might explain the improved transcytosis of SPN:Ply-low. Apart from these, the chain size also plays a role in dictating the fate in internalized SPN. Work by Iovino et al. (2016) demonstrates that, off the SPN, which exists mostly as chains in the bloodstream, a small fraction (<5%) of piliated, RrgA-expressing single-cocci are the ones that successfully cross the BBB. Although the identity and role of pneumococcal factors that enable evasion of BMEC intracellular defenses are becoming clearer, transit of the lysosome-evaded SPN-containing vacuole to the basal side of the polarized endothelium would require additional steps, probably involving manipulation of host cytoskeleton and motor molecules; this might be an interesting avenue for future research.

## Conclusion

**Figure 2:**
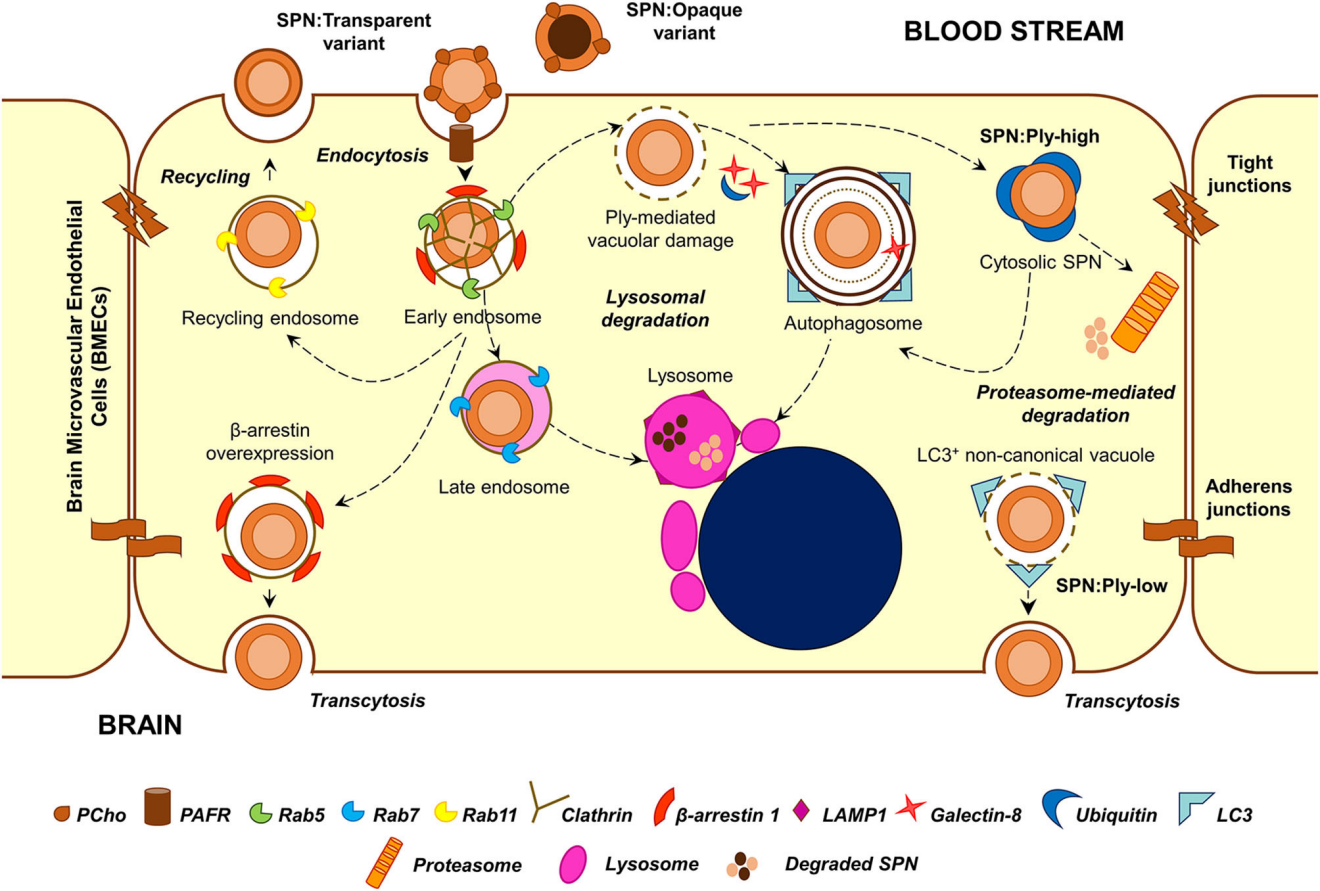
Major stages in the intracellular stint of SPN within BMECs. Following PCho-PAFR interaction, SPN is internalized into BMECs via clathrin-dependent endocytosis, in vacuoles decorated with β-arrestin 1. A major fraction of these vacuoles undergoes maturation and fusion with lysosomes; overexpression of β-arrestin 1 shunts these vacuoles away from lysosomal killing for improved transcytosis. A minor subset of these vacuoles recycles back to the apical side. Ply-mediated damage to SPN containing vacuoles trigger recruitment of cytosolic “eat me” signals, such as galectin-8 and ubiquitin, which target these vacuoles toward autophagic (xenophagic) degradation. Excessive damage to the vacuolar membrane enable SPN to escape into the cytosol, where it is tagged by ubiquitin for degradation by autophagy or proteasome-mediated pathway. Expression of low amounts of Ply also give rise to a unique Gal8^−^Ubq^−^LC3^+^ non-canonical autophagic vacuoles, which has been speculated to have improved transcytosis ability. The transparent phase variants of SPN, owing to higher amounts of surface-exposed PCho, demonstrate improved invasive, and transcytosis capability. On the other hand, a majority of the opaque phase variants are degraded within lysosomes.

Pneumococcal encounter with the BBB endothelium is a critical event in meningitis involving an interplay of several pneumococcal and host factors. Initial events of adhesion and invasion are driven by SPN surface proteins and involve usurping of diverse endothelial cell receptors for gaining entry into the cell (Figure 1). In spite of being an extracellular pathogen, SPN own impressive strategies to invade and evade the degradative machineries of the BBB endothelium for successful transcytosis (Figure 2). This involves utilizing specialized endocytic pathways that confer a survival advantage, maintenance of phenotypic variants in virulence factors, such as capsule and pneumolysin, which influences its intracellular fate, etc. Transcytosis across the capillary endothelium into the brain presents a niche for unrestricted pneumococcal replication, in turn, eliciting an overwhelming host inflammatory response, which significantly contributes to the tissue injury associated with meningitis. Current treatment for pneumococcal meningitis includes antibiotics and adjunctive therapy with corticosteroids, such as dexamethasone to manage the inflammation (Hoffman and Weber, 2009). Immunization with pneumococcal vaccines is an effective prevention strategy to reduce incidences of invasive pneumococcal diseases, including meningitis, but is complicated due to emergence and rise of non-vaccine serotypes (Hsu et al., 2009). Detailed understanding of the events involved in pneumococcal interaction with the BBB endothelium is hoped to enable better management of pneumococcal meningitis.

## Author Contributions

AA and AB wrote the manuscript and prepared the figures.

## Conflict of Interest

The authors declare that the research was conducted in the absence of any commercial or financial relationships that could be construed as a potential conflict of interest.

## References

[B1] Asmat T. M. Agarwal V. Saleh M. Hammerschmidt S. (2014). Endocytosis of *Streptococcus pneumoniae* via the polymeric immunoglobulin receptor of epithelial cells relies on clathrin and caveolin dependent mechanisms. Int. J. Med. Microbiol. 304, 1233–1246. 10.1016/j.ijmm.2014.10.001 25455218

[B2] Banerjee A. Van Sorge N. M. Sheen T. R. Uchiyama S. Mitchell T. J. Doran K. S. (2010). Activation of brain endothelium by pneumococcal neuraminidase NanA promotes bacterial internalization. Cell. Microbiol. 12, 1576–1588. 10.1111/j.1462-5822.2010.01490.x 20557315 PMC2943548

[B3] Bergmann S. Rohde M. Chhatwal G. S. Hammerschmidt S. (2001) α-Enolase of Streptococcus pneumoniae is a plasmin(ogen)-binding protein displayed on the bacterial cell surface. Mol. Microbiol. 40, 1273–1287. 10.1046/j.1365-2958.2001.02448.x 11442827

[B4] Bergmann S. Schoenen H. Hammerschmidt S. (2013). The interaction between bacterial enolase and plasminogen promotes adherence of *Streptococcus pneumoniae* to epithelial and endothelial cells. Int. J. Med. Microbiol. 303, 452–462. 10.1016/j.ijmm.2013.06.002 23906818

[B5] Collaborators G. B. D. M. (2018). Global, regional, and national burden of meningitis, 1990-2016: a systematic analysis for the global burden of disease study 2016. Lancet Neurol. 17, 1061–1082. 10.1016/S1474-4422(18)30387-9 30507391 PMC6234314

[B6] Cossart P. Helenius A. (2014). Endocytosis of viruses and bacteria. Cold Spring Harb. Perspect. Biol. 6:a016972. 10.1101/cshperspect.a016972 25085912 PMC4107984

[B7] Cundell D. R. Gerard N. P. Gerard C. Idanpaan-Heikkila I. Tuomanen E. I. (1995). *Streptococcus pneumoniae* anchor to activated human cells by the receptor for platelet-activating factor. Nature 377, 435–438. 10.1038/377435a0 7566121

[B8] Daneman R. Prat A. (2015). The blood-brain barrier. Cold Spring Harb. Perspect. Biol. 7:a020412. 10.1101/cshperspect.a020412 25561720 PMC4292164

[B9] Di Venanzio G. Lazzaro M. Morales E. S. Krapf D. Garcia Vescovi E. (2017). A pore-forming toxin enables serratia a nonlytic egress from host cells. Cell. Microbiol. 19:e12656. 10.1111/cmi.12656 27532510

[B10] Doran K. S. Banerjee A. Disson O. Lecuit M. (2013). Concepts and mechanisms: crossing host barriers. Cold Spring Harb. Perspect. Med. 3:a010090. 10.1101/cshperspect.a010090 23818514 PMC3685877

[B11] Florey O. Gammoh N. Kim S. E. Jiang X. Overholtzer M. (2015). V-ATPase and osmotic imbalances activate endolysosomal LC3 lipidation. Autophagy 11, 88–99. 10.4161/15548627.2014.984277 25484071 PMC4502810

[B12] Gradstedt H. Iovino F. Bijlsma J. J. (2013). *Streptococcus pneumoniae* invades endothelial host cells via multiple pathways and is killed in a lysosome dependent manner. PLoS ONE 8:e65626. 10.1371/journal.pone.0065626 23785439 PMC3681976

[B13] Hammerschmidt S. Wolff S. Hocke A. Rosseau S. Muller E. Rohde M. (2005). Illustration of pneumococcal polysaccharide capsule during adherence and invasion of epithelial cells. Infect. Immun. 73, 4653–4667. 10.1128/IAI.73.8.4653-4667.2005 16040978 PMC1201225

[B14] Hirose Y. Yamaguchi M. Goto K. Sumitomo T. Nakata M. Kawabata S. (2018). Competence-induced protein Ccs4 facilitates pneumococcal invasion into brain tissue and virulence in meningitis. Virulence 9, 1576–1587. 10.1080/21505594.2018.1526530 30251911 PMC6177246

[B15] Hoffman O. Weber R. J. (2009). Pathophysiology and treatment of bacterial meningitis. Ther. Adv. Neurol. Disord. 2, 1–7. 10.1177/1756285609337975 21180625 PMC3002609

[B16] Hsu H. E. Shutt K. A. Moore M. R. Beall B. W. Bennett N. M. Craig A. S.. (2009). Effect of pneumococcal conjugate vaccine on pneumococcal meningitis. N. Engl. J. Med. 360, 244–256. 10.1056/NEJMoa0800836 19144940 PMC4663990

[B17] Huotari J. Helenius A. (2011). Endosome maturation. EMBO J. 30, 3481–3500. 10.1038/emboj.2011.286 21878991 PMC3181477

[B18] Hyams C. Camberlein E. Cohen J. M. Bax K. Brown J. S. (2010). The *Streptococcus pneumoniae* capsule inhibits complement activity and neutrophil phagocytosis by multiple mechanisms. Infect. Immun. 78, 704–715. 10.1128/IAI.00881-09 19948837 PMC2812187

[B19] Iovino F. Engelen-Lee J. Y. Brouwer M. van de Beek D. van der Ende A. Valls Seron M.. (2017). pIgR and PECAM-1 bind to pneumococcal adhesins RrgA and PspC mediating bacterial brain invasion. J. Exp. Med. 214, 1619–1630. 10.1084/jem.20161668 28515075 PMC5461002

[B20] Iovino F. Gradstedt H. Bijlsma J. J. (2014). The proteasome-ubiquitin system is required for efficient killing of intracellular *Streptococcus pneumoniae* by brain endothelial cells. MBio 5, e00984–e00914. 10.1128/mBio.00984-14 24987087 PMC4161243

[B21] Iovino F. Hammarlof D. L. Garriss G. Brovall S. Nannapaneni P. Henriques-Normark B. (2016). Pneumococcal meningitis is promoted by single cocci expressing pilus adhesin RrgA. J. Clin. Invest. 126, 2821–2826. 10.1172/JCI84705 27348589 PMC4966305

[B22] Iovino F. Orihuela C. J. Moorlag H. E. Molema G. Bijlsma J. J. (2013). Interactions between blood-borne *Streptococcus pneumoniae* and the blood-brain barrier preceding meningitis. PLoS ONE 8:e68408. 10.1371/journal.pone.0068408 23874613 PMC3713044

[B23] Jen F. E. Warren M. J. Schulz B. L. Power P. M. Swords W. E. Weiser J. N.. (2013). Dual pili post-translational modifications synergize to mediate meningococcal adherence to platelet activating factor receptor on human airway cells. PLoS Pathog. 9:e1003377. 10.1371/journal.ppat.1003377 23696740 PMC3656113

[B24] Jimenez-Munguia I. Pulzova L. Kanova E. Tomeckova Z. Majerova P. Bhide K.. (2018). Proteomic and bioinformatic pipeline to screen the ligands of S. pneumoniae interacting with human brain microvascular endothelial cells. Sci. Rep. 8:5231. 10.1038/s41598-018-23485-1 29588455 PMC5869694

[B25] Kietzman C. C. Gao G. Mann B. Myers L. Tuomanen E. I. (2016). Dynamic capsule restructuring by the main pneumococcal autolysin LytA in response to the epithelium. Nat. Commun. 7:10859. 10.1038/ncomms10859 26924467 PMC4773454

[B26] King S. J. Hippe K. R. Weiser J. N. (2006). Deglycosylation of human glycoconjugates by the sequential activities of exoglycosidases expressed by *Streptococcus pneumoniae*. Mol. Microbiol. 59, 961–974. 10.1111/j.1365-2958.2005.04984.x 16420364

[B27] Luissint A. C. Artus C. Glacial F. Ganeshamoorthy K. Couraud P. O. (2012). Tight junctions at the blood brain barrier: physiological architecture and disease-associated dysregulation. Fluids Barriers CNS. 9:23. 10.1186/2045-8118-9-23 23140302 PMC3542074

[B28] Marra A. Brigham D. (2001). *Streptococcus pneumoniae* causes experimental meningitis following intranasal and otitis media infections via a nonhematogenous route. Infect. Immun. 69, 7318–7325. 10.1128/IAI.69.12.7318-7325.2001 11705903 PMC98817

[B29] Mook-Kanamori B. B. Geldhoff M. van der Poll T. van de Beek D. (2011). Pathogenesis and pathophysiology of pneumococcal meningitis. Clin. Microbiol. Rev. 24, 557–591. 10.1128/CMR.00008-11 21734248 PMC3131058

[B30] Ogawa M. Matsuda R. Takada N. Tomokiyo M. Yamamoto S. Shizukuishi S.. (2018). Molecular mechanisms of *Streptococcus pneumoniae*-targeted autophagy via pneumolysin, golgi-resident Rab41, and Nedd4-1-mediated K63-linked ubiquitination. Cell. Microbiol. 20:e12846. 10.1111/cmi.12846 29582580

[B31] Ogawa M. Takada N. Shizukuishi S. Tomokiyo M. Chang B. Yoshida M.. (2020). *Streptococcus pneumoniae* triggers hierarchical autophagy through reprogramming of LAPosome-like vesicles via NDP52-delocalization. Commun. Biol. 3:25. 10.1038/s42003-020-0753-3 31932716 PMC6957511

[B32] Oordt-Speets A. M. Bolijn R. van Hoorn R. C. Bhavsar A. Kyaw M. H. (2018). Global etiology of bacterial meningitis: a systematic review and meta-analysis. PLoS ONE 13:e0198772. 10.1371/journal.pone.0198772 29889859 PMC5995389

[B33] Orihuela C. J. Mahdavi J. Thornton J. Mann B. Wooldridge K. G. Abouseada N.. (2009). Laminin receptor initiates bacterial contact with the blood brain barrier in experimental meningitis models. J. Clin. Invest. 119, 1638–1646. 10.1172/JCI36759 19436113 PMC2689107

[B34] Radin J. N. Orihuela C. J. Murti G. Guglielmo C. Murray P. J. Tuomanen E. I. (2005). β-Arrestin 1 participates in platelet-activating factor receptor-mediated endocytosis of *Streptococcus pneumoniae. Infect. Immun*. 73, 7827–7835. 10.1128/IAI.73.12.7827-7835.2005 16299272 PMC1307033

[B35] Ring A. Weiser J. N. Tuomanen E. I. (1998). Pneumococcal trafficking across the blood-brain barrier. Molecular analysis of a novel bidirectional pathway. J. Clin. Invest. 102, 347–360. 10.1172/JCI2406 9664076 PMC508893

[B36] Sandoval K. E. Witt K. A. (2008). Blood-brain barrier tight junction permeability and ischemic stroke. Neurobiol. Dis. 32, 200–219. 10.1016/j.nbd.2008.08.005 18790057

[B37] Schnupf P. Portnoy D. A. (2007). Listeriolysin O: a phagosome-specific lysin. Microbes Infect. 9, 1176–1187. 10.1016/j.micinf.2007.05.005 17720603

[B38] Shizukuishi S. Ogawa M. Matsunaga S. Tomokiyo M. Ikebe T. Fushinobu S.. (2020). *Streptococcus pneumoniae* hijacks host autophagy by deploying CbpC as a decoy for Atg14 depletion. EMBO Rep. 21:e49232. 10.15252/embr.201949232 32239622 PMC7202210

[B39] Simionescu M. Popov D. Sima A. (2009). Endothelial transcytosis in health and disease. Cell. Tissue Res. 335, 27–40. 10.1007/s00441-008-0688-3 18836747

[B40] Surve M. V. Apte S. Bhutda S. Kamath K. G. Kim K. S. Banerjee A. (2020). *Streptococcus pneumoniae* utilizes a novel dynamin independent pathway for entry and persistence in brain endothelium. Curr. Res. Microbial Sci. 1, 62–68. 10.1016/j.crmicr.2020.08.001 PMC8610321 34841302

[B41] Surve M. V. Bhutda S. Datey A. Anil A. Rawat S. Pushpakaran A.. (2018). Heterogeneity in pneumolysin expression governs the fate of *Streptococcus pneumoniae* during blood-brain barrier trafficking. PLoS Pathog. 14:e1007168. 10.1371/journal.ppat.1007168 30011336 PMC6062133

[B42] Swords W. E. Ketterer M. R. Shao J. Campbell C. A. Weiser J. N. Apicella M. A. (2001). Binding of the non-typeable haemophilus influenzae lipooligosaccharide to the PAF receptor initiates host cell signalling. Cell. Microbiol. 3, 525–536. 10.1046/j.1462-5822.2001.00132.x 11488814

[B43] Tilley S. J. Orlova E. V. Gilbert R. J. Andrew P. W. Saibil H. R. (2005). Structural basis of pore formation by the bacterial toxin pneumolysin. Cell 121, 247–256. 10.1016/j.cell.2005.02.033 15851031

[B44] Yamaguchi M. Nakata M. Sumioka R. Hirose Y. Wada S. Akeda Y.. (2017). Zinc metalloproteinase ZmpC suppresses experimental pneumococcal meningitis by inhibiting bacterial invasion of central nervous systems. Virulence 8, 1516–1524. 10.1080/21505594.2017.1328333 28489958 PMC5810488

